# Impact of Sodium Zirconium Cyclosilicate Therapy Cessation in Patients with Systolic Heart Failure

**DOI:** 10.3390/jcm11185330

**Published:** 2022-09-10

**Authors:** Teruhiko Imamura, Nikhil Narang, Koichiro Kinugawa

**Affiliations:** 1 Second Department of Medicine, University of Toyama, Toyama 930-0194, Japan; 2 Advocate Christ Medical Center, Oak Lawn, IL 60453, USA

**Keywords:** chronic kidney disease, potassium, hyperkalemia

## Abstract

Background: Sodium zirconium cyclosilicate (SZC), a newly introduced potassium binder, is indicated for treating hyperkalemia. SZC-incorporated up-titration of renin-angiotensin system inhibitors and mineralocorticoid receptor antagonists has been recommended for those with systolic heart failure, whereas SZC is often terminated following the improvement of hyperkalemia in real-world practice. We aimed to investigate the impact of SZC cessation on the recurrence of hyperkalemia. Methods: Patients with systolic heart failure, in whom SZC was discontinued following improvement in hyperkalemia, were studied and compared to those who had continued SZC. All patients were followed for one year or until August 2022. The recurrent rates of hyperkalemia were compared between the two groups. Results: A total of 30 patients (median age 83 years, 53% men, median left ventricular ejection fraction 42%) were included. The one-year cumulative incidence of recurrent hyperkalemia was 93% in the group who discontinued SZC versus 22% in those who continued SZC (*p* = 0.032). In the group where SZC was withdrawn, doses of renin-angiotensin system inhibitors and mineralocorticoid receptor antagonists were less up-titrated, echocardiographic evidence of reverse remodeling occurred less, and readmission due to worsening heart failure tended to be higher compared to those who remained on SZC therapy. Conclusions: SZC cessation was associated with recurrent hyperkalemia and suboptimal medical therapy optimization compared to continuation of SZC therapy.

## 1. Background

Sodium zirconium cyclosilicate (SZC) is a recently introduced non-polymer zirconium silicate compound that decreases serum potassium levels by exchanging sodium and hydrogen for potassium and ammonium ions in the gastrointestinal tract [1]. Recent data support the feasibility of concomitant administration of SZCs to initiate/up-titrate heart failure-specific medical therapies including renin-angiotensin system inhibitors (RASIs) and mineralocorticoid receptor antagonists (MRAs), which commonly cause hyperkalemia and thus can be limited in their implementation [2]. Long-term SZC-incorporated medical therapy might theoretically improve clinical outcomes in patients with systolic heart failure by allowing the maximization and dose optimization of these foundational therapies. 

However, in real-world practice, potassium-binding agents are often utilized transiently as a rescue therapy until the hyperkalemia improves [3]. Given the high recurrence rate of hyperkalemia and associated incremental cardiovascular risks [4,5], the cessation of SZC might theoretically result in the recurrence of hyperkalemia and the termination/down-titration of RASI and MRA—potentially increasing the risk of worse clinical outcomes compared with long-term SZC-incorporating medical therapy. In this retrospective study, we investigated the association between SZC cessation and the recurrence of hyperkalemia in patients with systolic heart failure. 

## 2. Methods

### 2.1. Patient Selection

All patients with heart failure—which was diagnosed according to the Framingham’s criteria by the attending cardiologists—with a left ventricular ejection fraction (LVEF) <50% who received SZC between July 2020 and July 2022 to treat hyperkalemia (defined as serum potassium level >5.0 mEq/L) were retrospectively included. Patients dependent on hemodialysis or those taking sodium polystyrene sulfonate were not included. Informed consent was obtained from all participants and the present study was approved by the institutional ethical board beforehand.

### 2.2. Study Design

We assigned the included patients into two groups: those who discontinued SZC therapy (“Discontinue” group) and those who continued SZC therapy (“Continue” group). When SZC was terminated during the study period, patients were assigned to the discontinue group. The termination date was defined as day 0 and they were observed for one year or until the end of the study period. When SZC was continued for one year or until the end of this study, they were assigned to the continue group. Day 0 was defined as the time of SZC initiation in the latter group.

### 2.3. SZC Therapy

SZC was initiated to treat hyperkalemia at a loading dose for 2 days, in principle, followed by a maintenance dose of 5–15 g/day, according to the standard protocol. SZC was considered to be terminated either due to the improvement of hyperkalemia, serum potassium levels <3.5 mEq/L, or any other reason at the discretion of the treating physicians. 

### 2.4. Study Outcomes

The recurrence of hyperkalemia, which was defined as serum potassium levels >5.0 mEq/L, was the primary outcome of interest. The down-titration of RASIs and MRAs and the rate of readmission due to heart failure were the secondary outcomes of interest. 

### 2.5. Data Collection

Demographic, echocardiographic, laboratory, and medication data obtained at the time of SZC cessation (discontinue group) or at the time of SZC initiation (continue group) were retrieved as baseline characteristics. Doses of beta-blockers were stated as carvedilol equivalents. Doses of RASI—including angiotensin converting enzyme inhibitors, angiotensin receptor II blockers, and angiotensin receptor neprilysin inhibitors—were presented as enalapril equivalents. Doses of MRAs were presented as spironolactone equivalents. 

The recurrence of hyperkalemia, the dose of RASis and MRAs, and rate of heart failure readmissions were analyzed during the study period. Plasma B-type natriuretic peptide and transthoracic echocardiography data were obtained at 3 months follow-up if available.

### 2.6. Statistics

Statistics were conducted using SPSS Statistics 23 (SPSS Inc, Armonk, IL, USA). Two-tailed *p*-values < 0.05 were considered statistically significant. All variables were assumed as non-parametric data considering the small sample size. Clinical data were compared between the two groups: discontinue group versus continue group. Continuous variables were compared by Mann–Whitney U tests. Categorical variables were compared by Fischer’s exact tests. 

Trends in clinical parameters were compared using the Friedman test. Cumulative incidences were compared by the log-rank test. The prognostic impact of SZC secession upon the recurrence of hyperkalemia was investigated by Cox proportional hazard ratio regression analyses, which were adjusted for age and estimated glomerular filtration ratio. Event rates were compared between the two groups by negative binomial regression analyses. 

## 3. Results

### 3.1. Baseline Characteristics

A total of 30 patients with systolic heart failure (median age 83 years on median, 53% men) were included (Table 1). The median left ventricular end-diastolic diameter was 51 (48, 58) mm and the median LVEF was 42% (31%, 48%). The median plasma B-type natriuretic peptide was 254 (135, 316) pg/mL.

Of them, 21 patients terminated SZC due to normalization of hyperkalemia following a median 7 (3, 21) days of SZC therapy (Table 1). There were no other reasons for SZC termination. Their median age was 86 (79, 87) years old and 11 were men. At baseline when SZC was terminated (day 0), serum potassium levels were 4.7 (4.1, 5.1) mEq/L. The median doses of beta-blocker were 2.5 (2.5, 10) mg/day, RASIs were 2.5 (1.25, 2.5) mg/dL, and MRAs were 0 (0, 12.5) mg/day. 

The other nine patients who continued SZC for one year or until the end of this study were assigned to the control group (Table 1). Their median age was 78 (63, 78) years old and five were males. At baseline when SZC was initiated (day 0), serum potassium levels were 5.7 (5.3, 6.2) mEq/L. 

There were no statistically significant differences in the baseline characteristics between the two groups, except for a higher age and higher prevalence of ischemic heart disease in the SZC discontinue group (*p* < 0.05 for both; Table 1). According to the definition of each group (SZC was terminated on day 0 due to the improvement of hyperkalemia in the discontinue group; SZC was initiated on day 0 to treat hyperkalemia in the continue group), baseline serum potassium levels were significantly lower in the discontinue group (*p* = 0.001). 

### 3.2. Occurrence of Hyperkalemia (Primary Outcome)

**Figure 1:**
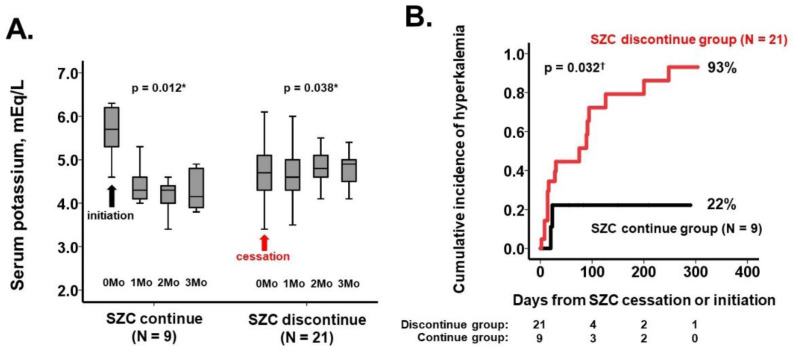
Trends of serum potassium levels for three months following the secession or initiation of SZC (**A**). One-year cumulative incidence of recurrent hyperkalemia in the SZC discontinue group and the SZC continue group (**B**). * *p* < 0.05 by Friedman tests for trend analyses. ^†^ *p* < 0.05 by log-rank test.

Following the cessation of SZC, serum potassium levels trended to increase again during the 3-month observational period (*p* = 0.038; Figure 1A). For 3 months following the initiation of SZC, serum potassium levels tended to decrease (*p* = 0.012; Figure 1A). 

During a one-year observational period, 16 patients in the discontinue group and two in the continue group encountered hyperkalemia. The cumulative incidence of hyperkalemia was higher in the SZC discontinue group (93% versus 22%, *p* = 0.032; Figure 1B). 

The cessation of SZC was an independent risk factor for the occurrence of hyperkalemia, with a hazard ratio of 4.691 (95% confidence interval 1.035–21.3, *p* = 0.045), adjusted for age and estimated glomerular filtration ratio.

### 3.3. Trends in Clinical Parameters (Secondary Outcomes)

**Figure 2:**
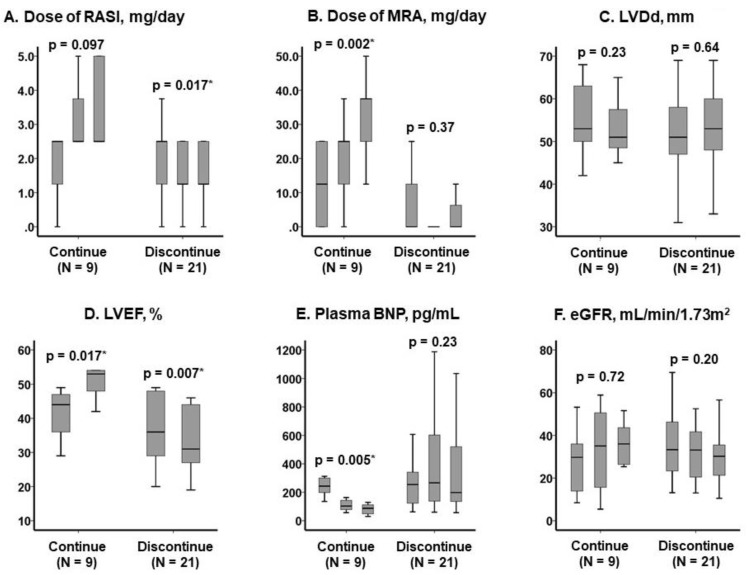
Trends of clinical variables including dose of RASIs (**A**), MRAs (**B**), LVDd (**C**), LVEFs (**D**), plasma BNP levels (**E**), and eGFRs (**F**) in the SZC continue group and the SZC discontinue group. Data were followed at baseline, 1 month, and 3 months later in medication doses and laboratory data (**A**,**B**,**E**,**F**). Data were followed at baseline and 3 months later for echocardiography data (**C**,**D**). * *p* < 0.05 by Friedman tests for trend analyses. RASI, renin-angiotensin system inhibitor; MRA, mineralocorticoid receptor antagonist; LVDd, left ventricular end-diastolic diameter; LVEF, left ventricular ejection fraction; BNP, B-type natriuretic peptide; eGFR, estimated glomerular filtration ratio.

Following the cessation of SZC, the doses of RASIs tended to decrease (*p* = 0.017), while MRA dosing remained unchanged (*p* = 0.37). The doses of RASIs remained unchanged (*p* = 0.097) and those of MRAs tended to increase (*p* = 0.002) following the initiation of SZC (Figure 2A,B). Prescription rates of SGLT2 inhibitors remained unchanged during the 3-month observational period both in the discontinue group (24%, 31%, and 31%, *p* = 0.54) and in the continue group (44%, 44%, and 43%, *p* = 0.76).

The left ventricular end-diastolic diameter remained unchanged in the two groups (*p* > 0.05 for both; Figure 2C). The LVEF decreased in the discontinue group (*p* = 0.007), whereas it tended to increase in the continue group (*p* = 0.017; Figure 2D). 

Plasma levels of the B-type natriuretic peptide remained unchanged in the discontinue group (*p* = 0.23) and decreased longitudinally in the continue group (*p* = 0.005; Figure 2E). The estimated glomerular filtration ratio tended to decrease in the discontinue group, whereas it improved in the continue group (*p* > 0.05 for both; Figure 2F).

### 3.4. Occurrence of Clinical Events (Secondary Outcomes)

Patients in the discontinue group had one all-cause death and five heart failure hospitalizations during the observational period. Patients in the continue group had one all-cause death and no heart failure hospitalization during the observational period. 

**Figure 3:**
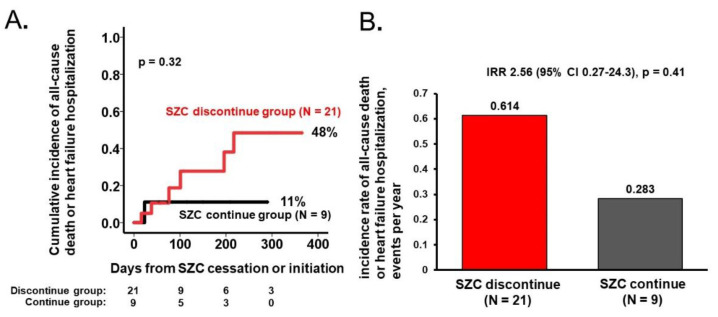
One-year cumulative incidence of all-cause death or heart failure hospitalization following SZC cessation/initiation (**A**). Incidence rate comparison between the SZC continue and SZC discontinue group (**B**). Log-rank test for time-to-event analysis and negative binomial regression analysis for incidence rate comparison. IRR, incidence rate ratio; CI, confidence interval.

The one-year cumulative incidence of clinical events tended to be higher in the discontinue group (48% versus 11%, *p* = 0.32; Figure 3A). The one-year event rate tended to be higher in the discontinue group (0.614 versus 0.283, incidence rate ratio 2.56, 95% confidence interval 0.27–24.3, *p* = 0.41; Figure 3B).

## 4. Discussion

In this retrospective analysis, we investigated the impact of the cessation of SZC—which was initiated to treat hyperkalemia in patients with systolic heart failure—by comparing its outcomes to those who continued SZC. SZC cessation was associated with a 93% 1-year recurrence rate of hyperkalemia, and also with less up-titration of RASI and MRA and reverse remodeling, along with a higher rate of readmission from heart failure. 

### 4.1. SZC Cessation and Recurrence of Hyperkalemia

Patients with systolic heart failure often experience hyperkalemia due to multiple risk factors including comorbid chronic kidney disease and the use of potassium-preserving heart failure-specific medications [6,7]. In the UK primary and secondary care data—including 84,210 heart failure patients—26% had hyperkalemia and 18% had recurrent hyperkalemia at 6-month follow-up [4]. Furthermore, a longer duration of hyperkalemia—as well as repeated hyperkalemia with a large variability in serum potassium levels—were associated with increased cardiovascular risk [5].

Thus, potassium-binding agents are emerging as important adjunctive therapies in managing hyperkalemia—particularly in patients with chronic heart failure [2] Nevertheless, potassium-binding agents are often terminated after the initial normalization of hyperkalemia. In the REVEAL-HK trial, approximately 70% of patients with heart failure terminated potassium-binding agents within a year, whereas approximately 40% of patients had persistent hyperkalemia at 1-year follow-up [3]. In our study, 21/30 (70%) patients used SZC short-term only as rescue therapy for hyperkalemia. Most of the patients encountered recurrent hyperkalemia within 1 year following the cessation of SZC, although they had achieved early normalization of hyperkalemia. Of note, we had no drug-related adverse events, including hypokalemia and peripheral edema. 

Serum potassium levels were maintained within the normal range in the majority of the SZC continue group, as demonstrated in the HARMONIZE trial and the J-LTS trial [8,9].

### 4.2. SZC Cessation and Doses of RASis and MRAs

RASIs and MRAs can increase serum potassium levels, which limits their implementation and dose optimization—particularly in patients with systolic heart failure with or without chronic kidney disease [7]; this may limit their efficacy in meaningfully reducing the risk of heart failure readmission and death from cardiovascular causes, given the failure to meet the doses achieved in landmark clinical trials. With data purporting the advantages of up-titrating RASIs and MRAs in patients with heart failure and hyperkalemia in reducing mortality and morbidity, [10,11] concomitant administration of SZC is highly recommended to maintain serum potassium and to avoid down-titration/discontinuation of life-saving medical therapies [2].

Nevertheless, as discussed above, SZC is often terminated when improvement of hyperkalemia is achieved. To manage recurrent hyperkalemia, doses of RASIs and MRAs often cannot be up-titrated. As a result, patients in this study had less evidence of reverse remodeling following SZC cessation. Probably due to persistent cardio-renal syndrome, their renal function did not improve during the study period. Although further prospective randomized-control studies are warranted to clarify robust causalities among those who receive SZC for longer durations, it would be recommended to continue SZC as long as possible even after the improvement of hyperkalemia in patients with systolic heart failure to maintain serum potassium levels and to up-titrate RASIs and MRAs to the target dose. 

### 4.3. Limitations

This is a proof-of-concept study, consisting of a small sample size due to the limited prescription of SZC to treat hyperkalemia in patients with systolic heart failure receiving RASIs and MRAs. Several non-significant differences in the two-group comparison might have reached statistical significance in larger-scale studies. Our findings should be validated in larger-scale multi-institutional randomized controlled studies. Although some variables did not reach statistically significant differences, the discontinue group was relatively sicker than the continue group; such background differences might have affected the clinical outcomes. The timing of SZC cessation and dose adjustment of medical therapies was determined at the discretion of the attending physicians. Thus, the detailed causality between SZC cessation, the recurrence of hyperkalemia, the dose adjustment of medical therapies, and clinical outcomes remains undemonstrated. In patients with more advanced heart failure, cardio-renal syndrome would facilitate hyperkalemia, hypotension, and renal dysfunction. Thus, the applicability of our findings to more advanced heart failure cohorts remains uncertain.

### 4.4. Conclusions

The cessation of SZC was associated with recurrent hyperkalemia, less up-titration of RASIs and MRAs, less cardiac reverse remodeling, and a higher rate of heart failure readmissions.

## Figures and Tables

**Table 1:** Baseline characteristics.

	Total (*n* = 30)	SZC Discontinue (*n* = 21)	SZC Continue (*n* = 9)	*p* Value
Demographics				
Age, years	83 (75, 87)	86 (79, 87)	78 (63, 78)	0.019 *
Men	16 (53%)	11 (52%)	5 (56%)	0.87
Body mass index	21.4 (18.9, 22.8)	21.7 (18.8, 23.0)	20.8 (18.9, 22.8)	0.86
Systolic blood pressure, mmHg	124 (116, 136)	122 (114, 138)	118 (116, 136)	0.66
Pulse rate, bpm	72 (65, 78)	70 (63, 82)	72 (64, 83)	0.59
New York Heart Association class (I/II/III/IV)	0/25/5/0	0/19/2/0	0/6/3/0	0.27
History of heart failure hospitalization	13 (43%)	9 (43%)	4 (44%)	0.94
Cardiac resynchronization therapy	2 (7%)	1 (5%)	1 (11%)	0.52
Hospitalized/ambulatory at day 0	16/14	12/9	4/5	0.52
Comorbidity				
Diabetes mellitus	16 (53%)	11 (52%)	5 (56%)	0.87
Atrial fibrillation	8 (27%)	4 (19%)	4 (44%)	0.15
Ischemic heart disease	16 (53%)	14 (67%)	2 (22%)	0.025 *
History of stroke	5 (17%)	5 (24%)	0	0.11
Echocardiography data				
Left ventricular end-diastolic diameter, mm	51 (48, 58)	51 (47, 57)	53 (50, 63)	0.42
Left ventricular ejection fraction, %	42 (31, 48)	37 (29, 48)	44 (36, 47)	0.19
Left atrial diameter, mm	43 (37, 46)	43 (37, 49)	43 (41, 45)	0.97
E/e’ ratio	13.3 (10.7, 16.0)	13.7 (11.5, 17.0)	11.5 (10.2, 15.2)	0.32
Mitral valve regurgitation mild or greater	6 (20%)	3 (14%)	3 (33%)	0.23
Tricuspid valve regurgitation mild or greater	2 (7%)	2 (10%)	0	0.34
Laboratory data				
Hemoglobin, g/dL	10.9 (9.7, 11.8)	10.3 (9.7, 11.8)	11.7 (10.6, 13.7)	0.21
Serum sodium, mEq/L	140 (137, 141)	140 (138, 141)	138 (135, 141)	0.26
Serum potassium, mEq/L	4.9 (4.4, 5.5)	4.7 (4.1, 5.1)	5.7 (5.3, 6.2)	0.001 *
Estimated glomerular filtration ratio, mL/min/1.73 m^2^	32.2 (20.3, 44.0)	32.9 (21.8, 46.6)	29.7 (13.9, 36.0)	0.28
Plasma B-type natriuretic peptide, pg/mL	254 (135, 316)	254 (123, 341)	243 (198, 300)	0.56
Medications				
Beta-blocker dose, mg/day	5.0 (2.5, 10.0)	2.5 (2.5, 10)	5.0 (2.5, 15)	0.14
Renin-angiotensin system inhibitors dose, mg/dL	2.5 (1.25, 2.5)	2.5 (1.25, 2.5)	2.5 (2.5, 2.5)	0.48
Mineralocorticoid receptor antagonists dose, mg/dL	0 (0, 12.5)	0 (0, 12.5)	12.5 (0, 25)	0.30
Furosemide dose, mg/dL	20 (0, 20)	20 (0, 20)	10 (0, 20)	0.65
SGLT2 inhibitor	9 (30%)	5 (24%)	4 (44%)	0.26

Continuous variables are expressed as median and interquartile and categorical variables are expressed as numbers and percentages. Continuous variables are compared by Mann–Whitney U test and categorical variables are compared by Fischer’s exact test. * *p* < 0.05.

## Data Availability

Data are available from the corresponding author upon appropriate request.
